# Meta-Analysis of Methamphetamine Modulation on Amyloid Precursor Protein through HMGB1 in Alzheimer’s Disease

**DOI:** 10.3390/ijms22094781

**Published:** 2021-04-30

**Authors:** Sedra Alabed, Heping Zhou, Ilker K. Sariyer, Sulie L. Chang

**Affiliations:** 1 Institute of NeuroImmune Pharmacology, Seton Hall University, South Orange, NJ 07079, USA; Alabedse@shu.edu; 2 Department of Biological Sciences, Seton Hall University, South Orange, NJ 07079, USA; Heping.zhou@shu.edu; 3 Department of Neuroscience and Center for Neurovirology, Temple University Lewis Katz School of Medicine, Philadelphia, PA 19122, USA; ilker.sariyer@temple.edu

**Keywords:** Ingenuity Pathway Analysis (IPA), APP, Aβ, METH, AD, HMGB1, blood brain barrier (BBB), network meta-analysis (NMA), neuroinflammation

## Abstract

The deposition of amyloid-beta (Aβ) through the cleavage of amyloid-beta precursor protein (APP) is a biomarker of Alzheimer’s disease (AD). This study used QIAGEN Ingenuity Pathway Analysis (IPA) to conduct meta-analysis on the molecular mechanisms by which methamphetamine (METH) impacts AD through modulating the expression of APP. All the molecules affected by METH and APP were collected from the QIAGEN Knowledge Base (QKB); 78 overlapping molecules were identified. Upon simulation of METH exposure using the “Molecule Activity Predictor” feature, eight molecules were found to be affected by METH and exhibited activation relationships on APP expression at a confidence of *p* = 0.000453 (Z-score = 3.51, two-tailed). Core Analysis of these eight molecules identified High Mobility Group Box protein 1 (HMGB1) signaling pathway among the top 5 canonical pathways with most overlap with the 8-molecule dataset. Simulated METH exposure increased APP expression through HMGB1 at a confidence of *p* < 0.00001 (Z-score = 7.64, two-tailed). HMGB1 is a pathogenic hallmark in AD progression. It not only increases the production of inflammatory mediators, but also mediates the disruption of the blood-brain barrier. Our analyses suggest the involvement of HMGB1 signaling pathway in METH-induced modulation of APP as a potential casual factor of AD.

## 1. Introduction

Alzheimer’s disease (AD) is a form of dementia characterized by the accumulation of amyloid beta (Aβ) plaques, neuronal damage and loss, neurofibrillary tangles of tau protein, and neuroinflammation. The deposition of Aβ monomers occurs through the proteolytic cleavage of amyloid precursor protein (APP), a transmembrane protein and one of the most abundant proteins in the central nervous system (CNS) [1]. The processing of APP can be non-amyloidogenic or amyloidogenic. The non-amyloidogenic pathway involves cleavage of APP by the enzyme α-secretase to produce a soluble *N*-terminal fragment, sAPPα, and a *C*-terminal fragment, which is then further cleaved by γ-secretase. In contrast, the amyloidogenic pathway involves cleavage of APP by β-secretase to produce a soluble *N*-terminal fragment, sAPPβ, and a C-terminal fragment, which is then further cleaved by γ-secretase to produce 40-amino-acid peptide Aβ40 and 42-amino-acid peptide Aβ42 [2]. Aβ peptides that are initially released as monomers progressively aggregate into oligomers and fibrils that mature to become Aβ plaques in the brain [3]. The accumulation of Aβ peptides into plaques gave rise to the “amyloid-β cascade” hypothesis that, although contentious, identifies these plaques as a central pathogenic feature of AD.

High Mobility Group Box 1 (HMGB1), a nuclear protein that interacts with DNA and histones and regulates transcription, has been implicated as a critical pathogenic hallmark in AD progression, in addition to Aβ and neurofibrillary tangles [4]. Aβ_25–35_-treated hippocampal neuronal cells exhibited increased HMGB1 expression in the AD-related model of neuroinflammation [5]. Aβ-induced microglial activation was also shown to upregulate HMGB1 and IL-1β, which may, in turn, further activate microglia and induce inflammation associated with AD [6]. HMGB1 may also serve as a damage-associated molecular pattern (DAMP) that activates the toll-like receptor (TLR)-4 signaling pathway outside the cells, leading to increased production of inflammatory mediators including cytokines and chemokines; promotes chemotaxis by forming a complex with chemokine C-X-C motif ligand (CXCL)12; or acts as a cytokine [7]. HMGB1 antibody inhibits neurite degeneration in the presence of Aβ plaques and recovers cognitive impairment in mice [8]. Inhibition of HMGB1 through oral pretreatment of glycyrrhizin was found to prevent postoperative cognitive impairment in aged mice [9], suggesting that HMGB1-mediated neuroinflammation may play a role in memory impairment. Exogenous HMGB1 has been shown to increase the accumulation of Aβ in microglial cytoplasm and inhibit the degradation of Aβ by microglia [10]. There is evidence that HMGB1 is increased in the extracellular fluid of AD brains [8]. Furthermore, HMGB1 is seen on the Aβ plaques in AD brains [10] and enhances the neurotoxicity of Aβ [11]. HMGB1 has also been found to mediate disruption of the blood-brain barrier (BBB) [12,13].

Methamphetamine (METH) is a potent psychostimulant drug with potential for psychological and physical dependence, due to its highly addictive nature [14]. METH abuse has become a worldwide issue in which 40% of global neuropsychological impairment has been found to be associated with its use [15]. Acute METH has also been shown to induce hyperthermia [16,17] and breakdown of the BBB [18]. Long term exposure to METH has been shown to be associated with neurodegeneration and cognitive impairment [19]. It induces damages to dopaminergic and serotonergic neurons, and activates microglia in the brain [20]. METH exposure decreases the immunoreactivity of dopamine transporter (DAT) in the striatum, function of serotonin transporter (SERT) in the hippocampus, and the content of serotonin (5-HT) in the frontal cortex of rodents [21]. Moreover, a recent clinical study found that amphetamine use increases the risk of developing dementia, including Alzheimer’s dementia by, fivefold [22]. These studies suggest that METH exposure may increase the risk for the development of AD. However, the molecular mechanisms are still unknown.

The growing body of literature associates neurodegeneration, neurotoxicity, and cognitive deficits caused by METH exposure with AD, and has raised concerns over significant public health problems caused by METH abuse in various communities across the United States [23]. However, the specific role of METH on the brain, whether beneficial or harmful, is still uncertain. Some studies have suggested that METH exhibits neuroprotective properties in low doses, particularly following traumatic brain injuries [24,25]; moreover, low doses of METH were found to partly influence the upregulation of α-secretase [26], which is critical for the production of sAPPα. sAPPα is a well-studied neuroprotective factor involved in enhancing long-term potentiation and memory [27]. In traumatic brain injuries, HMGB1 level is increased and contributes to the disruption of BBB integrity [12,13]. There have been very few studies examining the relationship between METH and HMGB1. One study reported that METH increases the striatal expression of HMGB1 [28]. However, the role of HMGB1 in METH-induced neuroinflammation remains unknown. Therefore, this study conducted network meta-analysis (NMA) to investigate the connections between METH and HMGB1, and to investigate the impact of METH exposure on the expression of APP through the HMGB1 pathway holistically. 

QIAGEN Ingenuity Pathway Analysis (IPA), a bioinformatics tool, was used for data mining and functional connectivity analysis of genes found in the QIAGEN Knowledge Base (QKB), a repository of manually curated information collected from over seven million individually modeled relationships between diseases, drugs, biological entities, such as genes, proteins, and metabolites, and processes that include expression, molecular cleavage, and phosphorylation, in addition to published results of omics experiments. In this study, the following tools were used: the “grow” tool expanded the molecules affected by METH, HMGB1, and APP; the “Path Explorer” tool connected the overlapping molecules between METH, HMGB1, and APP based on the expertly curated scientific literature stored in QKB; the “Molecular Activity Predictor” simulated the exposure of METH on the network to determine expression changes in HMGB1 and APP. Together, these tools were used to shed light on each individual connection between METH, HMGB1, and APP, while also providing an integrated and comprehensive network that sums the overall effects of those connections. Additionally, the Core Analysis feature and the canonical pathways were an integral part of this study as they aided in elucidating the role of not only every individual molecule, but how they may cause an impact when taken together—holistically. Finally, an algorithm, part of the “downstream effects analysis” identified by Kramer [29], was used to include quantitative feature of the studies by computing z-scores that reflect the directionality and magnitude of METH influence on HMGB1 and APP expression.

Molecules affected by METH and APP were obtained from QKB and the influence of METH exposure on APP-related molecules was characterized. Core Analysis of the eight molecules overlapped between METH and APP revealed HMGB1 signaling pathway as a top canonical pathway. Through the various tools of IPA, our network meta-analysis study suggests the involvement of HMGB1 as part of the METH-induced neuroinflammation as a potential casual factor of AD. This sheds light on potential mechanisms by which METH exposure contributes to the development of AD.

## 2. Results

### 2.1. Molecules Affected by METH and APP

**Figure 1:**
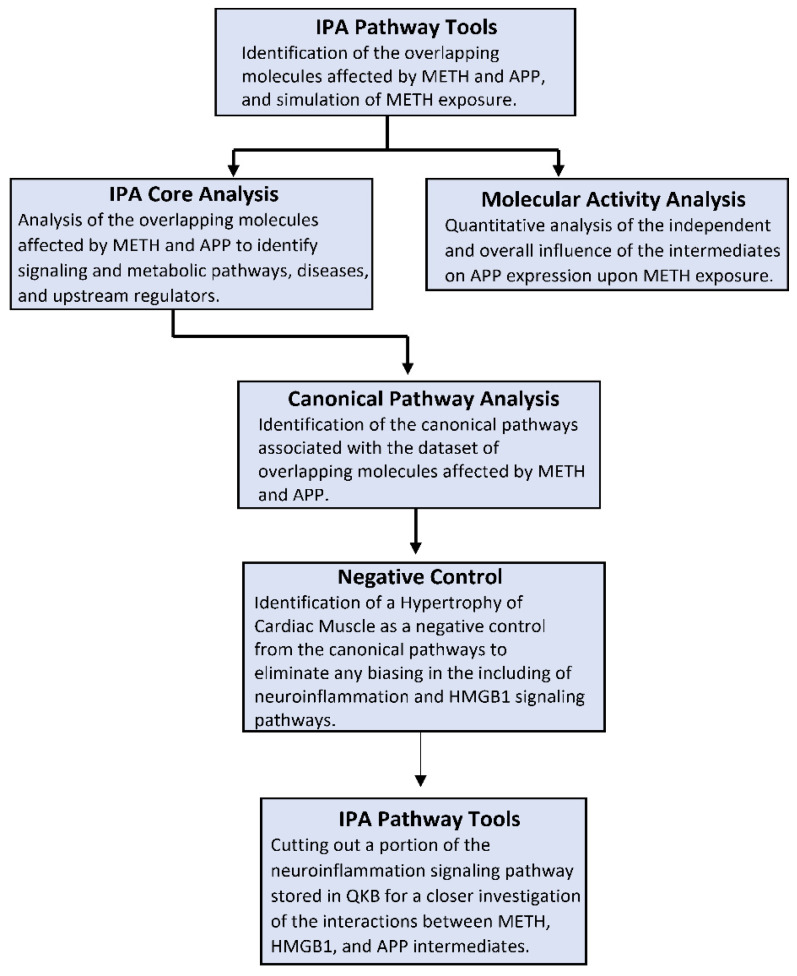
Methods Flowchart. The steps involved in utilizing the resources offered by IPA are outlined in the flowchart. The first step was to use the basic IPA pathway tools, including but not limited to “Grow”, “Trim”, “Connect”, “Pathway Finder”, and “MAP”. The molecular activity analysis was conducted after the generation of every pathway using mathematical algorithms and data stored in QKB. A core analysis was run on the dataset of molecules from every generated pathway; the resulting canonical pathways were analyzed to identify the role of the molecules within the canonical pathways. IPA tools were again used to excise a portion of the neuroinflammation signaling pathway stored in QKB for a closer and more detailed look of the implicated molecules.

**Figure 2:**
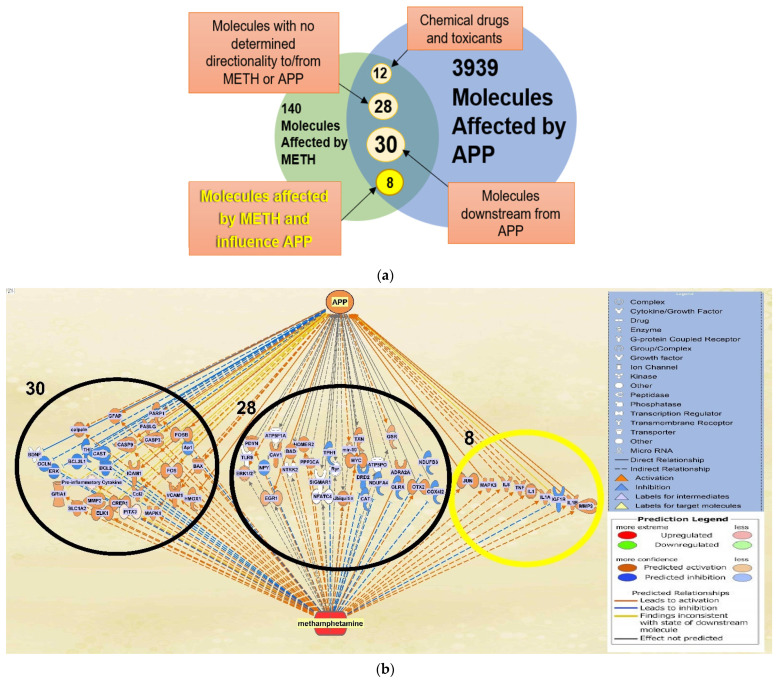
Categorization of the overlapping molecules. (**a**) The 78 overlapping molecules affected by METH and APP are categorized into groups to identify the molecules to be investigated further in the analysis, in which the impact of METH exposure influences APP expression; (**b**) the 78 overlapping molecules affected by METH and APP that were categorized into groups can be seen in clusters within the network (grey lines indicate an undetermined expression change) to offer an overview of the original overlapping network, sans the 12 chemical drugs and toxicants.

IPA’s “Build” and “Grow” tools were used to identify molecules affected by METH and APP, respectively, in QKB (Figure 1). A total of 140 molecules were found to be affected by METH and 3939 molecules were found to be associated with APP. A total of 78 molecules were found to overlap between molecules affected by METH and those associated with APP (Figure 2). Among these 78 molecules, 12 were chemical drugs and toxicants and thereby not included in further analysis in order to simulate biological systems; 28 molecules had either undetermined changes upon METH exposure or inconclusive directionality or influence on APP expression, and thereby were excluded from further analysis; 30 molecules were downstream of APP and could potentially have an effect on AD progression, but their influence is not contingent upon METH exposure, and therefore were excluded from further analysis since our study focused on the effects of METH on APP; and the remaining 8 molecules, JUN, MAPK3, IL6, TNF, IL1, IL1A, IGF1R, IL1B, and MMP9 affected by METH and upstream of APP were further analyzed. Among these 8 molecules, JUN, MAPK3, IL6, TNF, IL1, IL1A, IL1B, and MMP9 were activated by METH exposure and IGF1R was inhibited by METH exposure. Furthermore, expression of APP was increased by JUN, MAPK3, IL6, TNF, IL1, IL1A, IGF1R, IL1B, and MMP9, but mildly increased by IGF1R.

### 2.2. Quantitative Characterization of the influence of METH Exposure on APP Expression

The “Downstream Effect Analysis”, described by Kramer [29], uses an algorithm that determines the consistency of the expression and directional changes within a network of molecules; a Z-score is computed from the algorithm, yielding a value that reflects that consistency. The algorithm is facilitated by the use of prior biological knowledge and empirical scientific evidence derived from the repository of curated information stored in the QKB; the identified 8 molecules had a total of 166 references used as data points in the algorithm. This algorithm was used to quantify the extent of the impact of METH exposure on the intermediates and consequent expression change in APP; thus, it was used to examine the molecular mechanisms by which METH impacts the expression of APP and AD development. 

**Figure 3:**
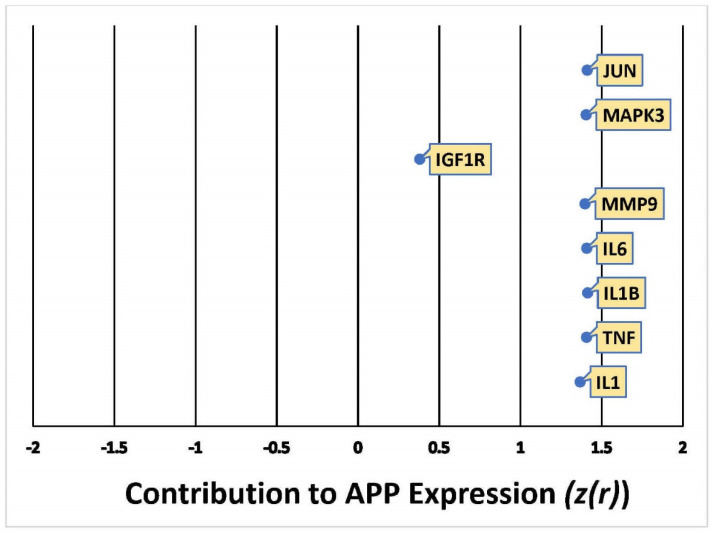
Quantitative illustration of the change in APP expression in response to METH exposure. The overall change in APP expression in response to METH exposure by the 8 intermediate molecules was measured by the individual involvement, Z-score (z(r)), of each one.

The cumulative z-score of METH impact on APP expression in the network was 3.5 [29,30], corresponding to a *p*-value of 0.000453 of a two tailed distribution; this indicates that the likelihood of finding the same results by chance occurs less than 1% of the time. The weights computed for the 8 molecules compiled in Figure 3 indicate the directionality of the change in APP expression, ranging from −2 (decrease) to +2 (increase), and the confidence of each expression change [29]. Out of the 8 molecules predicted to undergo activity changes upon METH exposure, JUN, MAPK3, MMP9, IL6, IL1B, TNF, and IL1 were predicted to upregulate APP expression with a Z-score of more than 1.5, while IGF1R was predicted to affect APP expression with a Z-score of less than 0.5. The overall impact of these 8 molecules upon METH exposure was predicted to activate or increase the expression of APP, thereby simulating a casual network where METH was an upstream regulator of intermediate molecules, which increase the production of APP.

### 2.3. Canonical Pathway Analysis of the Intermediate Molecules Dataset

**Figure 4:**
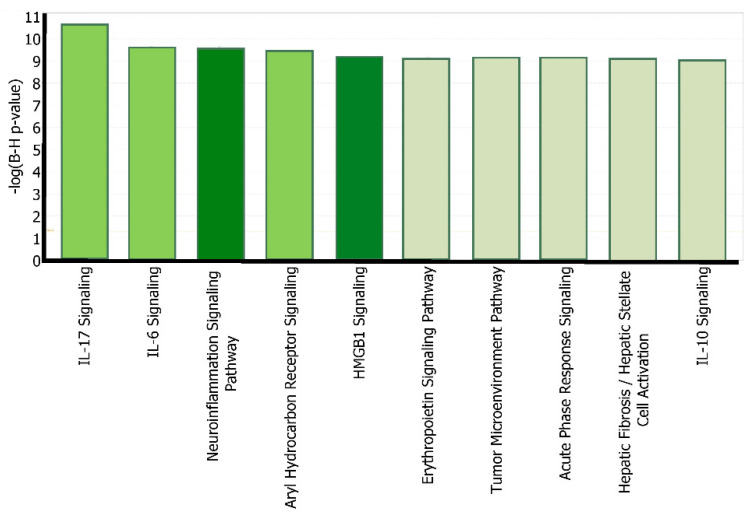
The core analysis of top canonical pathways. The core analysis of the 8 overlapping molecules (JUN, MAPK3, MMP9, IL6, IL1B, TNF, IL1, and IFG1R) identified a series of canonical pathways associated with the molecules, which are listed in order of decreasing –log(B-H *p*-value). The dark highlighted pathways, neuroinflammation signaling pathways, and HGMB1 signaling, were the ones chosen for further investigation of their role in Alzheimer’s disease.

“Expression” analysis was then conducted to compare these 8 intermediate molecules with the molecules that constitute each of the canonical pathways. These 8 molecules that influence APP expression following METH exposure were found to be involved in 296 out of 705 canonical signaling and metabolic pathways in IPA. Moreover, the top five canonical pathways, shown in Figure 4, with the highest significance are well-known to be involved in Alzheimer’s disease. Parts of the IL-17 and IL-6 signaling pathways are included in the HMGB1 and neuroinflammation signaling pathways, where IL-17 activates c-JUN and the MAPK cascade, and IL-6 activates c-JUN and ERK 1/2 cascade. The Aryl Hydrocarbon Receptor signaling pathway has been recently suggested to participate in the brain’s aging process and potentially neurodegeneration, as AHR serum levels were found higher in AD patients than others [31]. This study focused on the HMGB1 signaling pathway as a critical intermediate between METH and APP, and the neuroinflammation signaling pathway as a network that provides a holistic view of the overall impact of METH, HMGB1, APP, and their intermediates on neurodegeneration.

Due to the complexity of the immune system, there is a possibility that any core analysis run on any dataset would implicate the neuroinflammation and HMGB1 signaling pathways in the top canonical pathways. A clear bias favoring the neuroinflammation and HMGB1 signaling pathways as a canonical pathway in any given dataset would weaken the hypothesis of the impact of METH on APP through neuroinflammation and HMGB1. As such, from the top canonical pathways generated from the core analysis run on the dataset of the original 8 molecules, the 14th canonical pathway called, Cardiac Hypertrophy Signaling (Enhanced), with a *p*-value of 7.74 × 10^−11^, was chosen as a negative control as it is the highest canonical pathway that has no a-priori associations. A total of 115 molecules were found to overlap between Hypertrophy of Cardiac Muscle and APP. A core analysis of the dataset generated 388 canonical pathways out of the 705 stored in QKB. The neuroinflammation signaling pathway and HMGB1 signaling pathway were not among the top ten canonical pathways and none of the top 5 canonical pathways from the core analysis of the 8-molecule dataset overlapped with the top 5 canonical pathways from the cardiac hypertrophy with APP dataset. Therefore, the results of this negative control increased the confidence that identification of neuroinflammation and HMGB1 as the top of canonical pathways in our analysis was rather unique and not due to broad and non-specific associations.

### 2.4. The Integrated Network of Upregulated APP Expression through HMGB1 Following METH Exposure

**Figure 5:**
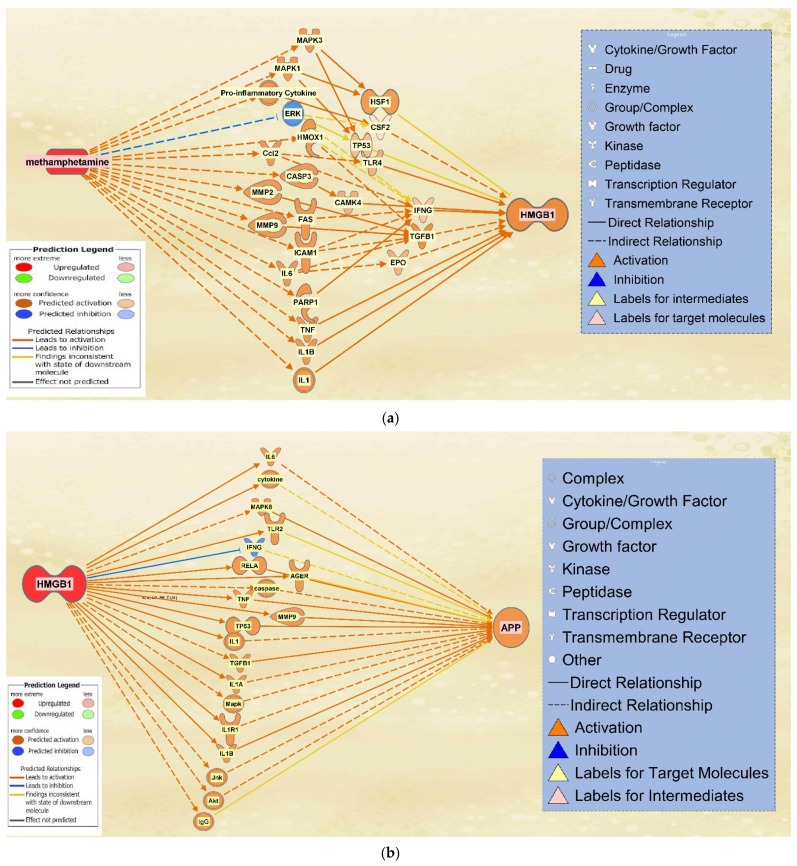
Overlapping Molecules Between METH and HMGB1 and APP. (**a**) 16 molecules were found to overlap between METH and HMGB1. These intermediates are affected by METH exposure and also influence HMGB1 expression; (**b**) 19 molecules were found to overlap between HMGB1 and APP. These intermediates influence APP expression upon the upregulation of HMGB1.

HMGB1 associated molecules were then identified from QKB using the “Build” and “Grow” tools. METH exposure was then simulated. Among the molecules affected by METH exposure and associated with HMGB1, 16 intermediate molecules were found to influence HMGB1 expression following METH exposure (Figure 5a). Similarly, molecules associated with both HMGB1 and APP were characterized. A total of 19 intermediate molecules were found to influence APP expression upon upregulation of HMGB1 (Figure 5b).

**Figure 6:**
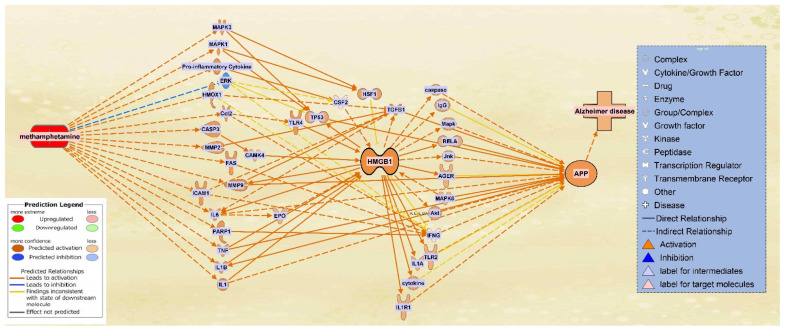
Integrated network of the METH, HMGB1, and APP intermediate molecules. The pathways between the 16 intermediate molecules influencing HMGB1 expression upon METH exposure and the 19 molecules mediating HMGB1 and APP were integrated into one network that illustrates the relationships of the molecules in the presence of each other. For a full list of molecules see Table 1.

A total of 16 molecules that were found to influence HMGB1 expression following METH exposure were then pooled together with the 19 molecules that were found to influence APP expression upon upregulation of HMGB1. METH exposure was simulated again to produce a comprehensive illustration of the changes in directionality, gene expression, and connections between the molecules within the network. The result was a network in which HMGB1 served as a primary intermediate between METH and APP, while APP maintained its position of being influenced downstream of the network (Figure 6).

**Figure 7:**
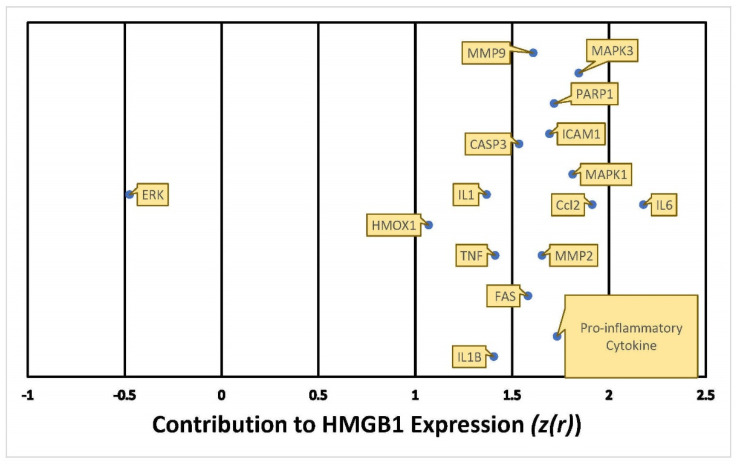
Quantitative illustration of HMGB1 expression in response to METH exposure. The overall change in expression in HMGB1 in response to METH exposure was measured by the individual involvement, Z-score (z(r)), of each of the 16 intermediate molecules.

In order to assign weights to the relationships of the molecules mediating HMGB1 and APP expression following METH exposure, Z-scores were calculated using the “Downstream Effect Analysis”, described by Kramer [29], based on an algorithm that determines the consistency of the expression and directional changes of the network with known scientific literature by computing a z-statistic. The algorithm used 420 references of prior known scientific literature stored in QKB as data points to predict how METH exposure may change HMGB1 expression. The Z-score for the involvement of each individual molecule on HMGB1 expression was calculated. As shown in Figure 7, among the 16 molecules that may affect HMGB1 expression, IL6 was found to affect HMGB1 expression with a Z-score higher than 2, 10 molecules with a Z-score between 1.5 and 2, and 4 molecules with a Z score between 1 and 1.5. ERK was found to affect HMGB1 expression with a Z-score close to −0.5 (Figure 7).

**Figure 8:**
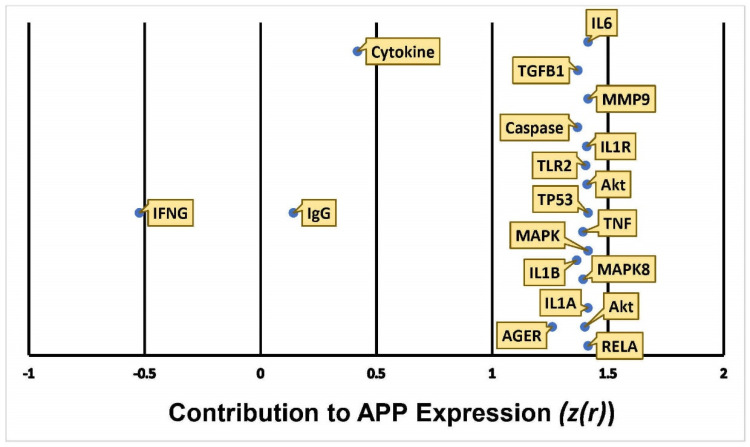
Quantitative illustration of APP expression in response HMGB1 upregulation. The overall change in APP expression following the upregulation of HMGB1 in response to METH exposure was measured by the individual involvement, Z-score (z(r)), of each of the 19 intermediate molecules.

The algorithm used 653 references of prior known scientific literature stored in QKB as data points to predict how HMGB1 upregulation may change APP expression. The Z-score for the involvement of each individual molecule on HMGB1 expression was calculated. As shown in Figure 8, 16 molecules affected APP expression with a Z-score between 1 and 1.5, 2 molecules with a Z score between 0 and 0.5; IFNG had a Z-score close to −0.5. 

The results indicated an increase in APP expression with an overall calculation that gave a Z-score of 7.6 [29,30], which corresponds to a *p*-value of less than 0.00001 in a two tailed hypothesis, suggesting that the likelihood of finding equally strong consistency of the results occurs at a less than 0.001% chance. 

### 2.5. The Effect of HMGB1 Inhibition on the Network

**Figure 9:**
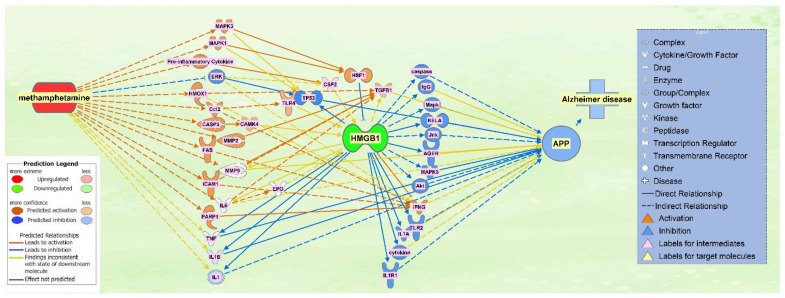
Activation of METH and Inactivation of HMGB1. METH was activated and HMGB1 was inactivated to illustrate the importance of the role of HMGB1 in METH influenced APP expression. The blocking of HMGB1 showed that even upon the exposure of METH, APP expression is inhibited.

To examine how critical the role of HMGB1 is in the network of METH and APP, the “MAP” tool was used to simulate how inhibition or blocking of HMGB1 may affect METH-induced modulation of APP expression. Figure 9 shows that blocking HMGB1 not only inhibited the activities downstream of HMGB1, but also inhibited molecules activated by METH including TNF, IL1B, and IL1. 

**Figure 10:**
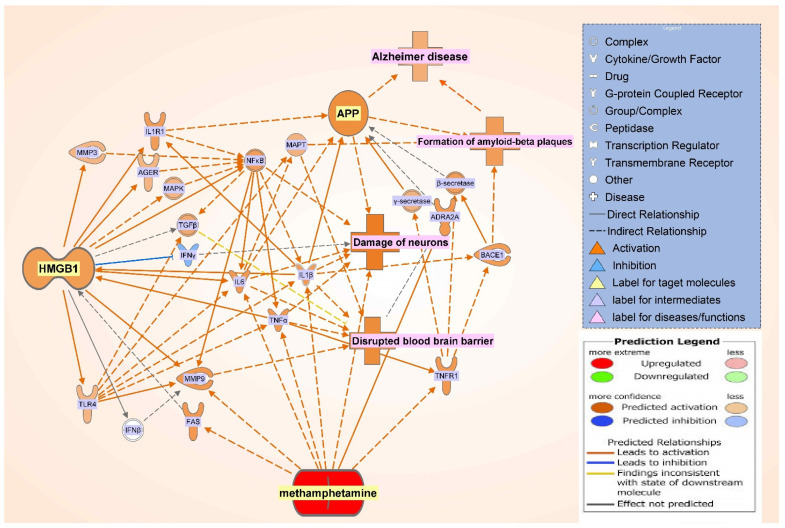
Canonical pathways of the METH, HMGB1, and APP integrated network. The core analysis of the integrated pathway revealed the canonical pathways associated with the network in order of decreasing −log(*p*-value), of which neuroinflammation signaling pathway was the top and selected for further investigation.

In addition, a core analysis was run on the dataset of the molecules in the integrated network of METH, HMGB1, and APP (Figure 6), which revealed the neuroinflammation signaling pathway to be of highest significance (*p*-value = 3.36 × 10^−31^). The pathway illustrated a variety of connections, of which TNF, IL1B, and IL6 are part of, that lead to neuronal damage, as well as disruption of the blood brain barrier, leading to neurodegenerative disease (Figure 10). The METH-induced release of these cytokines activated downstream signaling pathways, like HMGB1 pathway, that results in ultimate neuronal death or blood brain barrier disruption. In the investigation of the neuroinflammatory signaling pathway (Figure 10), nuclear factor-κB (NF-κB) was seen as a mediator that leads to the activation of the mentioned proinflammatory molecules, as well as a direct relationship with neural damage.

## 3. Discussion

This study examined how METH may increase in the expression of APP using network meta-analysis. Our meta-analysis found that METH increased expression of HMGB1, which, in turn, leads to disruption of the blood brain barrier (BBB), neuronal damage, formation of amyloid-beta plaques, and an overall increase in APP expression (Figure 10). These data were in line with prior findings on the relationship between HMGB1 and its effects on the BBB and APP [10,11,12,13,32,33] to demonstrate METH modulation on APP, the precursor of beta amyloid, through HMGB1 in AD development.

This study also sheds light on the controversy of whether METH exposure is harmful [15,17,34] or beneficial [24,25] to the nervous system. Our simulation showed that METH increased the activity of key inflammatory mediators, including IL6, TNF, IL1, IL1A, IL1B, and JUN, a transcription factor. These key inflammatory mediators increased the activity of APP through various molecular mechanisms (Figure 2b and Figure 6). The computed z-scores (Figure 3) provide a possible explanation for the conflicting data, as mentioned above [15,17,24,25,34], on whether METH exerts neurotoxic or neuroprotective effects on the brain. Figure 3 shows all 8 molecules to increase APP expression individually, although the impact of IFG1R was weaker. The understanding of the significance of the overall z-score is critical; the individual change in expression caused by IGF1R is less than 0.5, while the rest of the 8 molecules have z-scores close to 1.5. This means that taking into account only one molecule, such as IFG1R, would indicate that one molecule does not have a strong impact, nor can it provide a holistic perspective. The overall z-score for APP expression from Figure 3 was 3.5; corresponding to a *p*-value of 0.000453 of a two tailed distribution; this indicates that the likelihood of finding the same results by chance occurs less than 1% of the time. Thus, the 8 molecules presented in Figure 3, including IGF1R, and the molecules in Figure 6, provide a holistic perspective and confirm that METH has neurotoxic effects in the nervous system, as reported previously [15,17,34].

Our studies found that JUN is activated by METH and influences APP expression with a Z-score of 1.411 (Figure 3). Indeed, administration of METH has been reported to increase the JUN protein in the striatum and frontal cortex of mice, which is involved in METH-induced apoptosis in various parts of the brain. JUN has also been shown to increase the transcription of APP in human glial cells [35]. Moreover, the IL-17 signaling pathway—the most significant canonical pathway in Figure 4 (*p*-value = 7.88 × 10^−14^)—activates c-JUN [36,37], and the MAPK cascade [38,39]. METH has also been reported to induce the activation of microglia [34]. When microglia are activated, the production of TNF, IL1B, IL1A, and IL6 is significantly increased [40,41,42]. TNFα plays a key role in striatal dopaminergic neurotoxicity [42]. The increased immunoreactivity of IL1B, IL6, and TNF in microglia was detected in AD patients [43]. IL1B and TNF have been reported to stimulate Aβ production [44], which METH is also shown to induce [45]. Our studies found that increased production of APP by IL1B, IL6, and TNF may be mediated by c-JUN N-terminal kinase (JNK)-dependent MAPK pathway. This pathway is part of the IL-17 signaling pathway (Figure 3). IL1B, IL6, and TNF are also stimulators of γ-Secretase (seen activated in Figure 10), which cleaves APP.

Figure 6 shows the individual relationship between METH and ERK, which possibly sheds light on the neuroprotective properties of METH in low doses [25]. At 8 days following the treatment of METH, the phosphorylation of ERK was increased [46]. ERK cascade is involved in regulating long term potentiation, learning, and memory [47]. The computed z-score for ERK (Figure 7) is −0.5, suggesting an inhibitory effect on the expression of HMGB1, which suggests that METH may have neuroprotective properties. However, when ERK was considered together with the other molecules, the z-score becomes positive, which suggests that the protective effects of decreased ERK are mitigated and overpowered by other intermediates. Time course studies of METH exposure may be conducted to examine whether activation of ERK precedes the activation of the other molecules and pathways that induce neurotoxicity and neuroinflammation. 

Matrix metalloproteinase-9 (MMP9) was another overlapping molecule between those activated by METH and those influenced by APP. In rodents, repeated METH administration induces behavioral sensitization and an increase in MMP-2/9 activity in the brain [16,17,45]. When activated, MMPs are regulated by natural inhibitors—tissue inhibitors of matrix metalloproteinases (TIMPs). MMPs, which are known to function in extracellular matrix remodeling, have been implicated in numerous neuropathologies of neurodegeneration and neuroinflammation; molecules, such as TNF and IL6, require processing by MMPs or ADAMs for maturation, indicating that when MMPs levels are high, the up-regulation of these pro inflammatory cytokines significantly modulates neuroinflammation [48]. Moreover, MMPs, with the regulation of TIMPs, have been suggested to be involved in the activity-dependent reorganization of neuronal architecture [49]. Specifically, MMP9 is involved in the dendritic remodeling of the hippocampus of an adult rat. Administration of METH has led to behavioral sensitization, followed by the up-regulation of MMP-2, MMP-9, and TIMP-2 expression in various parts of the brain, including the frontal cortex, as well as an increase in the expression of MMP-2/9 in neurons and glial cells. The authors in the study suggest that the repeated use of METH induces overexpression of those molecules, leading to dendritic structural changes and subsequently, behavioral sensitization to METH [50]. As a mediator between METH and APP, HMGB1 regulates the expression of MMP9, where our simulation indicated an increase in MMP9 expression. The administration of an HMGB1 antagonist was found to decrease IL1-β mediated expression of MMP1/3/9 [51,52]. The expression of MMP9 is also regulated by HMGB1 through other molecules, including toll like receptor 4 (TLR4) [53]. MMP2 has been found to degrade Aβ at a much faster rate than MMP9, providing a route for Aβ clearance [54]. Increased expression and activation of MMP2/9 has been found to associate with Aβ deposits in parenchyma lesions in AD brain [55]. In our study, Figure 10 shows that MMPs may also lead to the disruption of the BBB, and the increased BBB disruption may enhance the production of APP, which may, in turn, lead to an increase in Aβ deposits.

Further analysis of the most significant canonical pathway generated from the dataset from Figure 6, the specific role of HMGB1 in the neuroinflammation signaling pathway was identified (Figure 10). Figure 10 shows the evidence of the role of HMGB1 is that when simulated, it initiates cytokine production through TLR4, thus inducing neurodegeneration [56]. Figure 10 shows HMGB1 as an upstream molecule for the cytokines mediating between it and APP; such molecules are IL1R and IL1A. In later studies, HMGB1 acts as a potent stimulator of monocyte cytokine synthesis (including IL1R and IL1A), with the exception of IL-10 and IL-12 [32]. Whereas pure HMGB1 was found to fail to induce or minimally induce pro inflammatory activity [57], another type of intermediates downstream from HMGB1 are Toll-like receptors (TLR) which were indicated to be involved in cellular activation through HMGB1; specifically, TLR2 and TLR4 interact with HMGB1 through TLR-dependent and independent pathways in the HMGB1 signaling pathway that activates cells [58]. The integrated pathway in Figure 6 showed TLR4 as a mediator between METH and HMGB1, while TLR2 was a mediator between HMGB1 and APP; however, the neuroinflammation signaling pathway in Figure 10 shows HMGB1 interacting as an upstream regulator for TLR4. Furthermore, Figure 10 shows TLR4 mediating the effects of HMGB1 with BBB disruption, neuronal damage, and activation of APP. Interestingly, the phosphorylation of MARCKS, a submembrane protein, is initiated prior to the Aβ aggregation in AD and before the onset of cognitive impairment in both human and mouse AD brains; it is also a hallmark for neuritis degeneration [59]. The trigger of the phosphorylation of MARCKS results from HMGB1 binding to TLR4. In a study, anti-HMGB1 monoclonal antibody was shown to delay the onset of AD before the aggregation of Aβ, as well as after; the authors suggested that suppressing the phosphorylation of MARCKS is the possible mechanism underlying HMGB1 involvement in AD onset [8].

Another mediator between HMGB1 and APP was receptor for advanced glycation end products (RAGE), which has been implicated in various neurodegenerative diseases, such as AD. RAGE has been found in AD brains at a higher concentration than normal brains. Going back to the blood brain barrier damage and its relation to AD, data suggests that RAGE carries Aβ across a leaky BBB [60]. Toxic Aβ peptides then can penetrate into, and deposit in, the central nervous system (CNS) [61,62,63]. HMGB1 has been recently associated with a damaged BBB and found in high concentrations following the activation of neuroinflammatory cytokines, representing itself as an early onset hallmark of AD [8,12]. Figure 4 shows that our Core Analysis has identified HMGB1 signaling pathway as one of the top 5 canonical pathways with the most overlap with the 8-molecule dataset affected by METH. This revealed HMGB1 as one of the molecular mechanisms by which METH leads to AD.

As any methodology, network meta-analysis has its limitations. This study identified 8 intermediate molecules that are affected by METH and influence the activity of APP using IPA and QKB. QKB has been generated using research findings published and curated so far. This study has focused on these 8 molecules. Our findings do not exclude the possible indirect involvement in METH actions or APP activities from other molecules that might be potentially associated with any of the 8 intermediate molecules. Secondly, IPA uses the over representation analysis (ORA), which evaluates the statistical relevance of a given pathway, is based on differentially expressed components within the pathway beyond the molecules that could be randomly expected. Every component in the pathway is assigned an equal weight, regardless of whether the component is inherent to the interactions. However, all of the 8 intermediate molecules between METH and APP consistently exhibited activating effects on APP with a cumulative Z-score of 3.5. These data confirm that these 8 molecules are the key molecules mediating the impact of METH on APP expression. Despite the limitations mentioned above, this study has revealed holistic molecular connections of METH activation leading to enhanced production of APP through HMGB1, shedding light on how METH may increase AD risk.

Altogether, this study examined the involvement of HMGB1 in METH-modulation on APP expression, where METH exposure was found to upregulate HMGB1 expression, which consequently increased the expression of APP. Blocking HMGB1 in the pathway led to inhibition in APP expression, suggesting that HMGB1 could potentially be a therapeutic target to reduce the accumulation of Aβ plaques in the brain and be effective in mitigating the risks of AD progression from METH use. Our study suggests that METH exposure may increase the expression of APP and that HMGB1 signaling pathway may play a key role in METH-induced expression of APP. Our data shed light on the potential mechanism by which METH exposure causes neurotoxicity and neurological deficits in AD, and that neuroinflammation, commonly found in AD brains, may mediate METH-induced APP expression through HMGB1. 

## 4. Materials and Methods

### 4.1. Ingenuity Pathway Analysis Software

The IPA Analysis Match CL license was purchased from QIAGEN and has been renewed annually since 2018 (QIAGEN Inc., Germantown, MD, USA, https://www.qiagenbioinformatics.com/products). IPA is a bioinformatics tool that was used to analyze data and biological processes using the QKB repository composed of over seven million individually modeled relationships to produce and analyze networks through known metabolic and signaling pathways (Figure 1). Data used for METH and APP analysis in this study was retrieved from 2 November 2020 to 20 January 2021. 

### 4.2. Identification of Overlapping Molecules Affected by METH, HMGB1, and APP

IPA’s “Grow” tool was used to identify genes, proteins, complexes, and chemicals affected by METH, HMGB1, and APP. The “Pathway Explorer” tool was used to add biological context to the molecules by connecting them through data in QKB. The “Trim” tool was used to eliminate molecules that do not occur in biological systems, such as chemical drugs or toxicants.

### 4.3. Connectivity and Molecule Activity Predictor (MAP)

The molecular relationships added through the “Grow” and “Pathway Explorer” tools were analyzed by simulating changes in activity processes, such as expression, transcription, activation, inhibition, or phosphorylation using the “MAP” tool. The response to METH exposure was simulated by using the “MAP” tool to illustrate its influence on its downstream molecules identified in QKB.

### 4.4. Canonical Pathway Analysis through the Core Analysis

A dataset of the molecules overlapping in each network (METH → APP; METH → HMGB1 → APP) was created and uploaded into IPA for “Core Analysis”. Canonical pathway analysis queries the dataset against the 705 canonical pathways stored in QKB. The significance for the association with each of the canonical pathways was calculated using a Benjamini–Hochberg Corrected Fisher’s Exact test to generate a −log(*p*-value). This allowed the identification of biological pathways that the molecules in each dataset are involved in.

### 4.5. Negative Control of the Canonical Pathways

A negative control was done to confirm that no biasing occurred in the results of the canonical pathways implicated in a particular dataset. Because biological systems are complex and reactive, some more than others, there is a possibility that any dataset would result in an overlap with certain canonical pathways. A bias favoring the inclusion of certain canonical pathways, in the case of this study, neuroinflammation and HMGB1 signaling pathways, would weaken the hypothesis that METH exposure influences the expression of APP through neuroinflammation and HMGB1.

Out of the top canonical pathways generated from the core analysis run on the dataset of the METH and APP overlapping molecules, a pathway with no a-priori associations was chosen. It was then expanded using the “Grow” tool and the molecules overlapping with APP were identified. A dataset was created, and a core analysis was run to generate canonical pathways, which were then compared to those in the positive control.

### 4.6. Quantitative Analysis of the Influence of METH Exposure on APP Expression

The “Downstream Effect Analysis” algorithm, identified by Krämer [29], was used to calculate the quantitative weight for the expression change simulated by the “MAP” tool based on the findings stored in QKB. The algorithm uses QKB references as data points to confirm a confidence for the change in APP expression upon METH exposure.

(1) $$s\left(e\right)=\mathrm{sgn}(\underset{f\epsilon F\left(e\right)}{\sum }\tilde{s}\left(f\right))$$

(2) $$w\left(e\right)=\frac{1}{N+1}\;\left|\underset{f\epsilon F\left(e\right)}{\sum }\tilde{s}\left(f\right)\right|$$

(3) O˜(r)≔{vϵR(r)|sR(r,v)≠0 ∧vϵD}And we define the activation Z-score asz(r)= ∑vϵO˜wR(r,v)sR(r,v)sD(v)(∑vϵO˜[wR(r,v)]2)12

The formula identified by Krämer was utilized to compute a Z-score for each intermediate downstream from METH and upstream of APP by calculating its individual involvement in APP expression. The scale of individual expression change caused by a molecule in a given pathway runs between −2 and 2, where −2 indicates a strong inhibitory relationship and 2 indicates a strong activation relationship. Each relationship is composed of at least one edge, *e*, that is in the middle of the cause-and-effect relationship—in other words, an intermediate. Each edge has findings, *f* ∈ *F(e),* stored in QKB upstream and downstream of it. *s(e)* identifies the overall sign of a particular direction from the edge, ranging from {−1, 0, 1}; *w(e)* identifies the weight of the edge, ranging from {0, 1}. Both formulas use the findings, *f*, plugged in as data points of −1 or 1. S_D_ is the sign of the upstream molecule in a given relationship as {−1, 1}. *z*(*r*) is then used to compute the individual change in expression of the target molecule in a relationship by combining the values computed for each direction downstream and upstream from a particular edge.

The same *z*(*r*) formula was used to compute an overall Z-score of a network of edges with a slight difference. W_R_, which corresponds to *w(e)* in the individual Z-score computation, now corresponds to *s(e),* which can be {−1, 1}. The same calculations were then performed.

Furthermore, another formula was used to compute an overall Z-score of a particular network combing all the individual Z-scores of each *edge* relationship. The formula is outlined by Stouffer and used to compute a Z-score aggregating independently found Z-statistics into a two tailed standard normal distribution [30,64].

(4) $$Z_{w}=\frac{\sum_{i=1}^{k}w_{i}Z_{i}}{\sqrt{\sum_{i=1}^{k}w_{i}^{2}}}$$

where *Z_S_* is the overall Z-score of the two-tailed standard normal distribution; *Z_i_* is the individual one tailed Z-score; and *k* is the total number of individual Z-scores in the standard normal distribution.

Both formulas yielded the same Z-scores to the nearest tenth.

### 4.7. Generation of the Neuroinflammation Signaling Pathway Featuring METH, HMGB1, APP, and Relevant Molecules and Diseases/Functions

The neuroinflammation signaling pathway that features METH, HMGB1, APP, intermediates, and other diseases/functions, such as disrupted BBB, neuronal damage, and Aβ accumulation, was generated using the pathway stored in QKB. The neuroinflammation signaling pathway, as part of the 705 canonical pathways stored in QKB, features a large number of molecules, diseases, and functions. The specific portion of the pathway that was overlapped with the majority of the molecules in our dataset was selected for closer investigation. Redundant intermediates were eliminated to focus on the interconnections of the molecules in our dataset and their roles in the neuroinflammation signaling pathway. METH was added to the network and the exposure of METH on the network was simulated using the “MAP” tool.

## Figures and Tables

**Table 1:** List of the 35 total molecule mediating METH, HMGB1, and APP.

Symbol	Entrez Gene Name	Location
AGER	advanced glycosylation end-product specific receptor	Plasma Membrane
Akt	Akt	Cytoplasm
CAMK4	calcium/calmodulin dependent protein kinase IV	Nucleus
CASP3	caspase 3	Cytoplasm
caspase	caspase	Cytoplasm
Ccl2	chemokine (C-C motif) ligand 2	Extracellular Space
CSF2	colony stimulating factor 2	Extracellular Space
Cytokine *	cytokine	Extracellular Space
EPO	Erythropoietin	Extracellular Space
ERK	Extracellular Receptor Kinase	Other
FAS	Fas cell surface death receptor	Plasma Membrane
HMOX1	heme oxygenase 1	Cytoplasm
HSF1	heat shock transcription factor 1	Nucleus
ICAM1	intercellular adhesion molecule 1	Plasma Membrane
IFNG	interferon gamma	Extracellular Space
IgG	immunoglobulin G	Extracellular Space
IL1	interleukin 1	Extracellular Space
IL6	interleukin 6	Extracellular Space
IL1A	interleukin 1 alpha	Extracellular Space
IL1B	interleukin 1 beta	Extracellular Space
IL1R1	interleukin 1 receptor type 1	Plasma Membrane
Jnk	c-Jun N-terminal kinase	Cytoplasm
Mapk	Mitogen-activated protein kinase	Cytoplasm
MAPK1	mitogen-activated protein kinase 1	Cytoplasm
MAPK3	mitogen-activated protein kinase 3	Cytoplasm
MAPK8	mitogen-activated protein kinase 8	Cytoplasm
MMP2	matrix metallopeptidase 2	Extracellular Space
MMP9	matrix metallopeptidase 9	Extracellular Space
PARP1	poly(ADP-ribose) polymerase 1	Nucleus
Pro-inflammatory Cytokine *	Pro-inflammatory cytokine	Other
RELA	RELA proto-oncogene, NF-kB subunit	Nucleus
TGFB1	transforming growth factor beta 1	Extracellular Space
TLR2	toll like receptor 2	Plasma Membrane
TLR4	toll like receptor 4	Plasma Membrane
TNF	tumor necrosis factor	Extracellular Space
TP53	tumor protein p53	Nucleus

* Indicates complex groups/complexes—their members will have the same predicted activity. Members of Pro-inflammatory Cytokine: CD40LG, CD70, CLCF1, CNTF, CSF2, CXCL8, Eda, EDA, FASLG, IFNG, IL11, IL12A, IL12B, IL13, IL15, IL17A, IL17B, IL17C, Il17d, IL17D, IL17F, IL18, IL1A, IL1B, IL1F10, IL2, IL21, IL25, Il3, IL3, Il31, IL31, IL33, IL36A, IL36B, IL36G, IL37, IL4, IL5, IL6, LEP, LIF, LTA, LTB, OSM, TGFB1, TGFB2, TGFB3, TNF, TNFSF10, TNFSF11, TNFSF12, TNFSF13, TNFSF13B, TNFSF14, TNFSF15, TNFSF4, TNFSF8, Tnfsf9, TNFSF9. Members of Cytokine:IL1A, IL1B, IL9.

## Data Availability

Not applicable.
